# Eating the elephant whole or in slices: views of participants in a smoking cessation intervention trial on multiple behaviour changes as sequential or concurrent tasks

**DOI:** 10.1186/1471-2458-12-500

**Published:** 2012-07-03

**Authors:** Preethi Koshy, Mhairi Mackenzie, Wilma Leslie, Mike Lean, Catherine Hankey

**Affiliations:** 1 Urban Studies, School of Social & Political Sciences/Institute of Health & Wellbeing, University of Glasgow, Glasgow, G12 8RS, UK; 2 Human Nutrition, School of Medicine, University of Glasgow, Glasgow, G4 0SF, UK

## Abstract

**Background:**

This paper explores smoking cessation participants’ perceptions of attempting weight management alongside smoking cessation within the context of a health improvement intervention implemented in Glasgow, Scotland.

**Methods:**

One hundred and thirty-eight participants were recruited from smoking cessation classes in areas of multiple deprivation in Glasgow and randomised to intervention, receiving dietary advice, or to control groups. The primary outcome of the study was to determine the % change in body weight. Semi-structured interviews were conducted with a purposive sample of 15 intervention and 15 control participants at weeks 6 (during the intervention) and 24 (at the end of the intervention). The current paper, though predominantly qualitative, links perceptions of behaviour modification to % weight change and cessation rates at week 24 thereby enabling a better understanding of the mediators influencing multiple behaviour change.

**Results:**

Our findings suggest that participants who perceive separate behaviour changes as part of a broader approach to a healthier lifestyle, and hence attempt behaviour changes concurrently, may be at comparative advantage in positively achieving dual outcomes.

**Conclusions:**

These findings highlight the need to assess participants’ preference for attempting multiple behaviour changes sequentially or simultaneously in addition to assessing their readiness to change. Further testing of this hypothesis is warranted.

**Trial Registration:**

ISRCTN94961361

## Background

The focus on preventive care in population health is widely documented as a response to a changing disease burden from infection to chronic illness that has occurred since the second half of the twentieth century [1,2]. Despite accumulated evidence that ill-health and health inequalities are structurally determined, a significant policy focus globally, and at individual country level, has been on three modifiable life-style risk factors for excess mortality and morbidity: poor diet, low levels of physical activity and smoking. Programmes aimed at tackling these different risk-enhancing behaviours have flourished in the last few decades and are commonly planned, funded, implemented and, results of their impact written about, in separate streams. However, because these behaviours are largely structurally determined, it is no surprise that they have a tendency to coexist, thus increasing the risk to individuals and communities of a plethora of chronic diseases [3-5]. This paper reports mainly on qualitative data gathered as part of a cluster-randomised controlled study which tested whether nutritional advice provided as an adjunct to group-based smoking cessation programmes could limit the weight gain associated with stopping smoking. It addresses the question of how intervention and control participants engaged in a behaviour change intervention view the prospect of making multiple changes.

### Background to multiple behaviour change

There is a lack of consensus in academic literature about whether multiple behaviour change interventions are effective and indeed, most lifestyle interventions are behaviour-specific. In the literature a single behaviour intervention can be one that is more accurately a single goal intervention that may be achieved through changing more than one behaviour. For example, weight loss may be achieved through both improved diet and increased physical activity. In this context multiple behaviour interventions are those that include separate goals that may not operate in tandem with one another. Interventions aimed at changing behaviours that share similar mechanisms are of interest as they offer the potential to act in concert with one another, thereby improving treatment impact and reducing health costs [6]. Some studies have detected co-variation of behaviour changes where a positive change in one behaviour pattern is accompanied by similar changes in other behaviours and this suggests that underlying mechanisms are not specific to a single behaviour. For example, research indicates that individuals who choose to improve activity levels also tend to adopt healthier eating habits and maintain smoking cessation as compared to those who do not [7-9].

Interventions underpinned by theoretical models of behaviour change have attempted to understand the process of behaviour change and the mediators that influence this transition. One example is the Transtheoretical model (TTM) which postulates that, based on their level of motivation, individuals may pass through the following five stages while changing behaviour: precontemplation (not considering change), contemplation (considering change), preparation (making small changes), action (actively making changes), maintenance (maintained changed behaviour for 6 months) [10]. While often referred to as the Stages of Change model, TTM is an integrative theoretical framework that also considers the role of psychological constructs such as self-efficacy and decisional balance in structuring outcomes [11,12]. In addition, interventions based on this model tend to take cognitive and behavioural process-oriented variables into account so that each participant receives tailored and stage-matched advice. A number of studies have shown that single behaviour change interventions modelled on this approach have had better outcomes than those which did not offer stage-matched advice [13,14].

Recently, significant changes in multiple behaviours have been reported following TTM-based interventions targeting different rubrics of behaviour such as smoking, high-fat diets and risky sun exposure [15]; physical activity, diet and lipid medication compliance [16]; stress and weight management [17]. This suggests that TTM may be an effective framework for multiple risk-behaviour interventions. Apart from interventions based on TTM, those based on social cognitive theory and social learning theory have also been successful at altering multiple behaviours [18,19].

Constructs specific to a behaviour are often considered to be the most effective drivers of change but some researchers suggest that by applying a more generalised construct such as ‘readiness to change’ or ‘concern for one’s health’, multiple behaviours can be modified [6,7,20,21]. Common mediating mechanisms of behaviour change such as self-efficacy, decisional balance and the use of cognitive and behavioural processes have been known to increase with progressive stages of change for different behaviours [8,22]. Thus, increase in participants’ self-efficacy as they transition from contemplation to action stages of one behaviour change, might increase their self-efficacy to make other changes. This suggests that specific behaviour changes should not be viewed as independent processes; instead, as sharing a level of integration.

Co-variation between changes in smoking, diet and physical activity patterns result in a myriad of potential outcomes for intervention studies. To take an example of a physiological pathway, adoption of physical activity may motivate individuals to reduce dietary fat [7]; another outcome may be that it limits weight concerns and hence encourages a successful quit attempt [8]. A contrasting psychological pathway may follow where unsuccessful efforts to reduce weight gain lower self-efficacy and decrease the likelihood of individuals engaging in smoking cessation programmes [23,24]. Equally, success in one outcome may have no impact on another [25,26].

Nonetheless, the decision to modify one risk factor presents an *opportunity* to modify others. Individuals already committed to smoking cessation may be more receptive to advice that promotes healthy eating and physical activity at this juncture for a variety of reasons. For some, receiving such advice may allay their concerns about post-cessation weight gain [23,24,27-29] and for others, their efforts to quit smoking may be part of an attempt to improve their overall health. Conversely, success in improving dietary and physical activity habits could serve as a gateway to smoking cessation, which some participants may perceive as a more difficult behaviour change, by increasing their motivation and self-efficacy [20].

Studies which included *weight control* components with *smoking cessation* interventions have however, had varying success. Hall et al. [30] and Pirie et al. [31] found that participants were unsuccessful at both changes. Perkins et al. [32] reported better cessation rates following a combined intervention but participants were unable to simultaneously limit weight gain.

In relation to combined *smoking cessation* and *physical activity* interventions, it has been suggested that cognitive mechanisms underlying both types of change are similar [22,33]. However, a systematic review of 13 studies that encouraged both *physical activity* and *smoking cessation* revealed that only one successfully encouraged cessation and improved exercise capacity at 12 months [34]. The reasons for these findings are unclear. Some researchers argue that if combinations of behaviours (such as smoking cessation and weight management) in an intervention are not complementary, they may place a behavioural burden on participants and competing interests may cause participants’ efforts to be unfocussed [7,30]. However, this is contrary to recent evidence which suggests that neither behavioural change is undermined in such interventions [35].

Efforts have been made to identify whether sequencing behaviour change interventions are likely to facilitate multiple positive outcomes but here too the results are ambiguous; some studies support the use of a sequential approach [35], others advocate the use of a simultaneous intervention [26] and a recent study by Vandelanotte et al. [36] was unable to detect any difference between the two approaches. Furthermore, there is no consensus on the optimum number of behaviours that individuals can change at a given time [37].

While multiple behaviour interventions offer a means of improving health using integrative paradigms, much remains to be understood about designing interventions that can effectively modify multiple risk factors [38]. Understanding how individuals perceive multiple behaviour change is crucial to ensuring the success of such interventions but this area remains unexplored. The aim of this paper is to explore whether participants recruited from smoking cessation classes and randomised to receive dietary advice, or to control groups, perceived these behavioural changes as linked or discrete processes.

## Methods

### Participants and setting

Smokers are known to have poorer diets than non-smokers [39] and weight gain following smoking cessation is a concern to both smokers wishing to quit smoking and policymakers. In an attempt to address these challenges, in 2007, a weight management intervention informed by the Transtheoretical Model was delivered to smoking cessation participants from deprived areas of Glasgow over 24 weeks; its components are detailed elsewhere [40] but it is important to note here that participants in the intervention arm were not explicitly directed whether behaviour change should occur sequentially or concurrently. The study aimed to determine whether a stage-matched intervention delivering dietary advice to smoking cessation participants would minimise post-cessation weight gain to ≤3% [39] and improve smoking cessation maintenance compared to controls receiving routine cessation advice. This cluster randomised study was approved by the West of Scotland Research Ethics Committee on 18th September 2007. The unit of randomisation was a smoking cessation class.

The primary outcome of the study was the difference in % weight gain between baseline and week 24 between intervention and control groups. A realist approach to evaluation of the intervention was adopted to understand the context in which specific mechanisms (for example, psychological mechanisms) were triggered among intervention and control participants that influenced weight management and smoking cessation outcomes [41]. The details of how a realist approach was applied are provided elsewhere [42].

### Interviews

**Figure 1:**
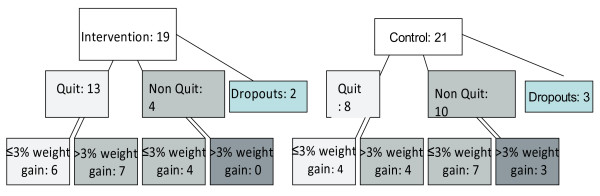
Numbers (%) attaining intervention goals among interviewed participants.

To explore participants’ perceptions and experiences of multiple behaviour change and to identify the mechanisms that triggered these, both groups were interviewed at weeks 6 and 24. At week 6, 15 pairs of participants were purposively sampled and matched for body mass index (BMI) and reported cigarette consumption at recruitment. Of these 30 participants, 2 intervention participants and 3 controls dropped out of the study and two participants – one control and one intervention participants were unable to attend interviews at week 24. So at week 24, 15 pairs of participants were re-sampled after matching them for BMI at recruitment. Thus, in total 19 intervention participants and 21 control participants were interviewed as shown in Figure 1. Of these, 12 intervention participants and 11 control participants were interviewed at week 6 and week 24. The distributions of age, gender, BMI and cigarette consumption levels among interviewed participants in both groups were similar (Table 1). Most participants were interviewed by the researcher at the class venue; where this was not possible, a telephone interview was carried out.

**Table 1:** Characteristics of participants interviewed at weeks 6 and 24

	Intervention	Control
Gender		
Male	7	7
Female	12	14
Age		
30–40	3	2
41–50	9	10
51–60	2	4
61–70	4	6
>70	2	1
BMI		
Normal weight	6	6
Overweight	5	5
Obese	5	7
Morbidly obese	3	3
Cigarette consumption		
<20	7	8
20–30	10	10
>30	2	3

### Analysis

The interviews were semi-structured, audio-taped with the participants’ consent, transcribed verbatim and entered into ATLAS-ti software [43]. Coding and analysis of transcripts were carried out according to emergent and *a priori* themes (the latter selected to help understand participants’ perceived mechanisms of change) (Table 2). These were linked to develop explanations. All transcripts were double-coded by PK and MM. During this process, emerging themes and explanations were discussed and agreed by the research team and as these developed we revisited transcripts to ensure that we had not missed concepts that contradicted these.

**Table 2:** ***A priori*and emergent themes**

*A Priori* themes	Emergent themes
· Psychological mechanisms for change related to the TTM and to the proven efficacy of group interactions	· Vicious and virtuous circles of change
· Physiological mechanisms relating to negative impacts of eating as a replacement to smoking and to positive impacts of improved respiratory function on physical activity	· Psychological mechanisms relating to awareness of eating as a replacement to smoking
· Health behaviours as integrated or discrete; sequential or simultaneous	

## Results

The quantitative results of this intervention are discussed in detail elsewhere (40). Statistical analyses showed that there were no significant differences in % weight change and cessation rates between the intervention and control groups. Furthermore, no significant changes to physical activity and eating habits were noted in either group at 24 weeks. These results, however, do not reveal how variations in context, delivery mechanisms and participant motivation can influence outcomes.

Nineteen intervention [19I] and twenty-one control [21C] participants were interviewed as illustrated in Figure 1. Of these, 13 intervention participants and 8 controls quit smoking but only 6 intervention participants and 4 control participants were also successful at limiting weight gain to ≤3% (Table 1).

The results are discussed under three main themes: diet, physical activity and smoking as separate behaviours to be tackled sequentially; diet, physical activity and smoking as part of an overall health change; and, the potential for multiple behaviours to operate in vicious and virtuous circles of change.

### Diet, physical activity and smoking as separate behaviours to be tackled sequentially

Of forty participants (19 intervention and 21 control participants) interviewed during the intervention at 6 weeks and/or at the end of the intervention at 24 weeks, around a quarter [3I, 8C] viewed diet, physical activity and smoking as discrete processes that could be most effectively changed sequentially rather than simultaneously. Unsurprisingly this view was more common within the control arm of the study since they had less explicit exposure to advice about managing simultaneous change but it was not exclusive to this group. The rationale for sequencing change (Table 3) was that changes were perceived to be easier to make when broken down into manageable chunks and different types of behaviour change were characterised as separate tasks. Nonetheless, holding this view did not prevent participants, in either control or intervention group, from achieving the goals of the intervention. Of the 21 quitters interviewed, 6 described a preference for changing behaviours sequentially; of the 10 who were successful at both quitting and restricting weight gain, three participants were of this view. Of the 11 participants interviewed who stated a preference for a sequential approach, 6 quit (1I, 5C) and 3 were successful at both changes (1I,2C).

**Table 3:** Illustrations of diet, exercise and smoking as separate behaviours to be tackled sequentially

Theme	Illustrative quotations
*Important to take one task at a time*	49(I): I feel that if I really push myself … I’m frightened I might just resort to having a cigarette instead … I’d rather that [smoking cessation] is done and dusted. [Q, <3%, 24th week]
	56(C): I don’t see the point in doing all this exercise when I’m still smoking anyway, so I’d rather nip that [smoking cessation] in the bud. [NQ, >3%, 24th week]
	44(C): Smoking was the last thing to deal with, I’ve dealt with the drinking, dealt with the weight, dealt with the smoking. [Q, <3%, 24th week]

Q – quitter; NQ – non-quitter; 6th week – 6th week interview; 24th week – 24th week interview.

Across those who viewed optimal change as happening either sequentially or concurrently more than a third [8I, 10C] described their desire to ‘treat’ themselves to comfort food to compensate for ‘depriving’ themselves of cigarettes during their quit attempt (see Table 4). These early perceptions of the difficulty in achieving more than one goal were not predictive of poor outcomes. At 24 weeks, 11 interviewed participants who quit smoking and four of those successful at both changes were from this group and all of them described changes they had made to their diet and physical activity alongside their quit attempt after realising that they were replacing cigarettes with food. Of the 18 interviewed participants who identified that they were replacing cigarettes with food, 13 quit (6I, 7C) and of these, 5 (2I, 3C) were successful at managing their weight.

**Table 4:** Illustrations of diet, exercise and smoking as replacement behaviours

Theme:	Illustrative quotations
Inevitability of eating filling space left by smoking	57(C): It’s your metabolism, so you are eating more, you are compensating for a cigarette and you’re not compensating with an apple or a banana or an orange. [Q, <3%, 24th week]
	46(I): Maybe you think ‘I’ll have a cup of tea and biscuit’ but I don’t want to do that so now it’s back to a wee cup of tea and cigarette. I need to be doing something, one or other. [NQ, <3%, 24th week]

Q- quitter; NQ-non-quitter; 6th week – 6th week interview; 24th week – 24th week interview.

### Concurrent behaviour change

Eating healthily, improving physical activity and quitting smoking were described as facets of an integrated healthy lifestyle change by 17 of those interviewed (10I, 7C). In the interviewed group a half of all quitters and slightly less than half of those who quit smoking and managed their weight believed that simultaneous behaviour change could be attempted effectively. While quitters who claimed to attempt behaviour changes simultaneously were not universally successful at weight management, they all described positive changes they had made to their diet and physical activity at 24 weeks. Of the 17 participants who viewed each of the changes as aspects of a healthy lifestyle change, 11 (8I,3C) quit smoking and of these 4 (2I,2C) were also able to manage their weight successfully. Thus, the concurrent approach was favoured by 11 quitters; 7 quitters preferred the sequential approach. Furthermore, 4 participants successful at both behaviour changes preferred the concurrent approach as compared to 3 who favoured sequential behaviour changes.

A number of different reasons were given for the view that concurrent behaviour change was desirable (see Table 5 for illustrative quotations). First, a small number of participants in both intervention and control groups noted that they were particularly keen to avoid post-cessation weight gain because of concerns that this would undermine any potential health benefits of smoking cessation. Second, around 16 of those interviewed stated that they viewed smoking, diet and physical activity patterns as inextricably linked. Third, three interviewees who successfully quit and restricted weight gain, expressed the view that a multiple behaviour change focus would be beneficial in diluting attention from each individual difficult shift. Thus, of seven participants who felt that attempting to improve their diet and physical activity could distract them from smoking and hence aid their quit attempt, 5 (3I,2C) quit and all 3 intervention participants were also able to limit weight gain to ≤3%.

**Table 5:** Illustrations of diet, exercise and smoking as connected behaviours best tackled concurrently

Theme:	Illustrative quotations
No desire to replace one set of illnesses with another	12(I): It’s one thing cutting out the cigarettes to cut out heart disease, but you’re just going to kill yourself anyway if you’re eating all these fatty foods. [NQ, <3%, 6th week]
	65(I): There’s no point in improving one side of your health to let the other side deteriorate. [Q, >3%, 24th week]
	21(C): I don’t want to [think] I’ve done one healthy thing and then all of a sudden I’m obese. [Q, <3%, 6th week]
Difficult to separate out individual behaviour changes	2(I): I’m willing to do anything to better my life from stopping smoking and eating healthily … and getting some form of exercise – Now, the three of them go hand in hand, don’t they. [Q, <3%, 6th week]
	35(C): Everything works in as one thing, you know, your not smoking, your eating healthy food, your on a control diet or whatever it is and your exercising – it’s not just four different things. [Q, <3%, 6th week]
Focus on multiple behaviours reduces focus on one alone	18(I): My daughter says ‘you don’t think it’s a bit much to focus on the two at the same time’ but I find it’s actually quite good because it takes my mind [off]. If I’m thinking about one, I’m not thinking about the other. [Q, <3%, 6th week]
	23(C): I think it would take your mind off thinking about cigarettes – you’ve got something else to think about and to focus on, so you’re not going [to] be focusing on cigarettes all the time. [Q, <3%, 6th week]

Q- quitter; NQ-non-quitter; 6th week – 6th week interview; 24th week – 24th week interview.

Participants who viewed multiple changes as more effective described both physiological and psychological synergies as accruing (see Table 6). In terms of physiological pathways, 10 interviewees [8I, 2C] discussed physiological mechanisms emanating from smoking cessation such as: improved breathing which encouraged them to be more active; and, better taste perception which helped them appreciate subtler tastes such as those of vegetables and fruit. Thus, of the 10 interviewed participants who identified physiological mechanisms as aiding behaviour changes, 7 (5I,2C) quit smoking and 3 (2I,1C) were successful at both changes.

**Table 6:** Implicit pathways to change

Theme:	Illustrative quotations
Physiological mechanisms	9(I): Fantastic. I just like the taste … when I was smoking and you were eating a piece of fruit it just tasted the same kind of bland … this time it’s really lovely … your carrot and your broccoli and all the different things that I’m eating … it’s just a lovely taste. [Q, >3%, 6th week]
	20(C): Now you can taste what it’s supposed to taste like. [Q, >3%, 6th week]
	47(I): My exercises – I can do a lot more because I’m not breathless, I can go up and down the stairs no bother. I just feel a lot healthier, a lot fitter and healthier. [Q, >3%, 24th week]
Psychological mechanisms	61(C): You know hard physical exercise makes you feel good and … healthy eating makes you feel good … and the smoking got to be a no no. [NQ, <3%, 24th week]
	9(I): I feel more refreshed and raring to go and I look forward to the day. [Q, >3%, 6th week]

Q- quitter; NQ-non-quitter; 6th week – 6th week interview; 24th week – 24th week interview.

A similar proportion [6I, 4C] identified improvements to their diet, physical activity and smoking as motivating them to maintain or make more positive changes. These participants felt that the psychological benefits they perceived while making one behavioural change encouraged them to make other healthy changes. Of the 10 interviewed participants who described psychological processes as encouraging behaviour changes, 6 (4I, 2C) quit and 3 of them (1I, 2C) were successful at managing their weight as well. These processes centred on improved confidence and increased self-efficacy induced by positive changes.

### Vicious and virtuous circles

The physiological and psychological pathways described above were also reflected in the ways that participants discussed behaviour changes as operating in both vicious and virtuous circles (see Table 7). Ten interviewed participants interviewed described such cyclical pathways. On one hand, three participants all of whom gained >3% weight described how perceived failure in one behaviour change triggered failure in others. Two of these participants were also unsuccessful at smoking cessation. On the other hand, six participants (3I,3C) described ‘virtuous’ pathways whereby achievements in one behavioural sphere would trigger success in others and of these 4 (2I,2C) quit smoking and 3 (1I,2C) of them were also successful at weight management. The pivotal role of each behaviour change in triggering change in others is aptly illustrated by the experience of one participant who in the 6th week described a positive cycle of multiple behaviour change which was subsequently stalled and reversed by a smoking relapse.

**Table 7:** Connectedness of behaviour change

Theme:	Illustrative quotations
Vicious circles	67(C): When I never had a cigarette I had this in my head … your lungs start clearing … inside me is getting cleared out so I’ll watch what I’m putting in … I was drinking plenty of water, fruit, vegetables, exercise every day and when I went back on the cigarettes, to hang with it, I started eating, fruit and veg is out the window and I’m back to the old sort of style [and] I’m actually smoking more. [NQ, >3%, 24th week]
	3(I): I’m angry at myself [for over-eating] – I shouldn’t be giving in to it – I’m actually worrying about it and, you know, it’s making me want to smoke … it’s a vicious circle really. [Q, >3%, 6th week]
Virtuous circles	65(I): If you’re eating the right stuff and you see yourself not putting on weight and feel fitter it gives you that extra gee to get up in the morning, your chest’s clear, you’ve not got a smoker’s cough, overall you feel better. [Q, >3%, 24th week]
	34(I): It [smoking cessation] is making me want to go to the gym and get my fitness back – if you’re feeling fit and you’ve got it in your mind that you’ve given up the smoking and you’re putting all the good nutrients into your body, well, aye, that would drive me on. [DO, 6th week]

Q- quitter; NQ-non-quitter; 6th week – 6th week interview; 24th week – 24th week interview.

## Discussion

While multiple behaviour change interventions are recognised for their potential to modify multiple behaviours and hence dramatically lower an individual’s risk of chronic disease, attempts are still being made to identify how various intervention components can be ordered to encourage behaviour change effectively. One criticism of previous multi-component studies has been that participants were not offered the opportunity to choose an approach to multiple behaviour change but in this study participants were able to attempt behaviour change in a manner they perceived as being feasible in the context of their own lives [44].

Our findings showed that whilst sequential behaviour change was the preferred approach among a quarter of interviewed participants (most of whom were controls), almost half of interviewed participants opted to simultaneously change their smoking, diet and physical activity patterns. Furthermore, participants who attempted behaviour change concurrently were more successful at quitting smoking and more successful at both behaviour changes as compared to those who attempted changes sequentially. This is supported by Hyman et al. who found that simultaneous attempts at behaviour change were more effective than sequential attempts. As in our study Hyman et al. noted that following sequential interventions, for most participants, success was likely to be restricted to a single behaviour change [26]. It should also be noted that a simultaneous multi-behaviour change study by Johnson et al. revealed that weight loss was achieved only after 24 months and was the outcome of changes to several behaviours [16]. This indicates that while concurrent approaches may initially appear to produce changes in a single behaviour, small changes in other behaviours may cumulatively produce beneficial outcomes over a longer period. Thus, participants in this study who were attempting to limit weight gain to ≤3% while quitting smoking may require a longer period to achieve weight stability as compared to individuals solely attempting to limit weight gain [39].

In contrast to our findings, Spring et al. identified sequential approaches to multiple behaviour changes as being superior to concurrent approaches [35]. This may have been because unlike other studies, intervention participants were offered intensive support including pre-packaged meals for 16 weeks which may have contributed to these results. However, it is worth noting that of those who opted for a sequential approach in our study more than half were successful at smoking cessation. This indicates that participants’ readiness to attempt behaviour change either sequentially or simultaneously may be a crucial factor in the behaviour change process.

Multiple behaviour change interventions modelled on TTM assess participants’ readiness to change and accordingly provide stage-matched advice which has been found to aid significant behaviour change. Our findings point to the need for also assessing participants’ readiness to attempt multiple behaviour changes either sequentially or simultaneously. Such an approach would take into consideration the contextual variations between individuals and allow them to engage in multiple behaviour change in an order that they perceive as making multiple behaviour change attainable. It is theoretically plausible that interventions which are tailored to individuals’ ‘readiness to change’ and ‘preferred approach to behaviour change’ may be more effective in aiding transition to action/maintenance stage across multiple behaviours [37]. Furthermore such interventions may be particularly effective in engaging hard-to-reach groups who may otherwise find multiple behaviour change untenable within the context of their lives [45].

Our findings also revealed that some mechanisms influenced multiple behaviour change regardless of whether participants adopted simultaneous or sequential approaches. For example some participants described their concern on realising that they were replacing cigarettes with food. However, for most participants this compensatory behaviour did not adversely affect their quit attempt. It is likely that for these participants, their desire to quit smoking and to avoid excessive weight gain despite food cravings may have been sufficient incentive to motivate them to make other positive changes to their diet and physical activity even though most did not limit weight gain to ≤3%. This is consistent with Nigg et al’s suggestion that certain individuals view multiple behaviour change through a hierarchical lens which determines the order and the extent to which they change behaviour at a given time and in response to specific incentives [37].

Other mechanisms that were identified as encouraging multiple behaviour change were psychological and physiological mechanisms. Psychological mechanisms such as improved confidence and increased self-efficacy following one behaviour change and physiological mechanisms such as improved taste sensation, ease of breathing and feeling ‘healthier’ were described as motivating participants to make further changes. These findings are consistent with those of other studies which indicate that one positive behaviour change can trigger physiological and psychological mechanisms that in turn encourage other behaviour changes [46-48].

The interplay of these mechanisms on multiple behaviours was evident in participants’ descriptions of how perceived success or failure in one behaviour change triggered vicious or virtuous circles of behaviour change. This highlights the need for identifying promising mechanisms that may trigger virtuous circles of behaviour change as well as the importance of understanding how vicious circles of behaviour change are triggered so as to design interventions that can effectively mediate these and thereby encourage multiple behaviour change [46].

This study offers a unique insight into the perceptions of participants’ attempting changes to their diet and physical activity alongside smoking cessation; to our knowledge no other study has explicitly explored this outwith the context of cardiac rehabilitation. It is worth mentioning that participants in this study were not informed that successful weight management was defined as limiting weight gain to ≤3%. As a result their goals and views of successful weight management may have been influenced by their body image, social norms, perceptions and experiences of weight gain and dieting during prior quit attempts. However, by not placing a target weight range, participants may have felt less pressured and more able to make changes to their diet and physical activity and maintain smoking abstinence despite post-cessation weight gain.

A limitation of this study was that the data revealed the perceptions and experiences of smokers in Glasgow who were attempting multiple behaviour change but it could not include the perceptions of those who dropped out of the study. It is important to explore the perceptions of this sub-group of the population in future studies as they are harder to engage in preventive services and may perceive multiple behaviour change differently. A second limitation is that, whilst the intervention took place in areas of multiple deprivation, data on the socio-economic status of participants were not collected and so we cannot determine the impacts of individual levels of poverty on participation in the study, outcomes achieved or perceptions of change. This is important because of the structural determinants of lifestyle behaviours, health outcomes and health inequalites.

## Conclusion

While the dietary intervention did not demonstrate a significant difference in % weight gain and cessation rates between intervention and control groups, it provided valuable insights into how participants attempted and perceived multiple behaviour change. Adopting a realist approach to evaluation of multiple behaviour change interventions thus enabled a better understanding of the combinations of contexts, type of participants and delivery mechanisms that were likely to influence behaviour change outcomes. Our findings indicate that participants who attempted behaviour changes simultaneously were more likely to succeed at one or more changes as compared to those who preferred a sequential approach. It also suggests that offering participants interventions that are stage-matched as well as matched to their preferred approach of behaviour change i.e, simultaneous or sequential interventions, may aid multiple behaviour change. The results also indicate that physiological and psychological mechanisms which participants perceived as strongly influencing behaviour change were not restricted to either approach. Finally, the paper demonstrates how the use of realist approaches to evaluation can illuminate the relationship between process and outcomes through the development and testing of mini-theories about what mechanisms trigger which changes in particular contexts. Refining our theoretical understanding in this way can help in designing multiple behaviour change interventions that deliberately encourage mechanisms that produce favourable outcomes while curbing those that produce undesirable outcomes. Consistent with the realist approach further research is needed to purposefully test these emerging findings and hence refine our understanding of how the potential for multiple behaviour change can be increased.

## Competing interests

The authors declare that they have no competing interests.

## Authors’ contributions

CH, ML, WL, MM and PK designed the study. PK carried out the fieldwork and analysis of the primary data. MM and PK wrote the preliminary version of this manuscript and all authors contributed to the final version. All authors read and approved the final manuscript.

## Pre-publication history

The pre-publication history for this paper can be accessed here:

http://www.biomedcentral.com/1471-2458/12/500/prepub
